# Preparing medical graduates to care for geriatric patients: A case study of the undergraduate medical curriculum at a South African university

**DOI:** 10.4102/safp.v62i1.5081

**Published:** 2020-04-20

**Authors:** Keshena Naidoo, Jacqueline van Wyk

**Affiliations:** 1 Discipline of Family Medicine, College of Health Sciences, University of KwaZulu-Natal, Durban, South Africa; 2 Department of Clinical and Professional Practice, College of Health Sciences, University of KwaZulu-Natal, Durban, South Africa

**Keywords:** medical education, health professions education, geriatric, elderly, curriculum

## Abstract

**Background:**

Medical schools in South Africa must be responsive to the health needs of the rapidly ageing population. Reports of the poor quality of care received by elderly patients raises concerns about the training of medical students. A review of the curriculum can help to assess current geriatric care training and identify the areas in need of improvement. This study was conducted to describe the nature and scope of undergraduate medical education in geriatric care at a South African university.

**Methods:**

An exploratory, descriptive case study was conducted to analyse the learning objectives, opportunities and outcomes of the 6-year undergraduate medical program. Data included an electronic curriculum supported by student and teacher guides. Semi-structured interviews were conducted with health professions educators.

**Results:**

The curriculum covered key geriatric competencies that included addressing geriatric syndromes and conducting a comprehensive geriatric assessment. Teaching on geriatric competencies occurred mainly in the clinical years, was integrated and no sub-minima was applied in its assessment. Teaching occurred in disciplinary silos with little involvement of the multidisciplinary team. Learning objectives and assessments focussed on geriatric knowledge and skills.

**Conclusion:**

The curriculum targets the development of student geriatric knowledge and skills, but not student attitudes towards caring for older patients. However, a national curriculum will ensure greater coverage of geriatric care competencies, particularly advocacy and attitudes towards caring for geriatric patients. Greater engagement with stakeholders in geriatric health care will inform suitable educational guidelines for undergraduate medical education in geriatric care at this institution. This may also contribute to a standardised national curriculum.

## Background

Health care professionals in South Africa are facing a rapidly ageing population with increased demands for age-related health care services.^1^ Old age is associated with chronic illnesses and functional impairments that result in an increased burden of disease among older adults.^2^ The high prevalence of multi-morbidities and sensory impairments in older adults requires an integrated approach from health care professionals with a focus on function and quality of life.^3^ The National Health Insurance Bill, which aims to provide Universal Health Coverage (UHC) to all South African citizens, is dependent on health professionals’ ability to deliver comprehensive health services to all at primary care level.^4^ It is thus essential that all medical students receive training in the core competencies of geriatric care to ensure the delivery of quality primary care services to elderly patients.^5^ The World Health Organization (WHO) highlighted the necessity of including geriatric training in undergraduate (UG) medical curricula almost two decades ago, but medical schools have been slow to implement this recommendation.^6^

Geriatric medicine, the field of medicine that deals with ageing and health conditions associated with advancing age, is a relatively new and neglected area in health professions education.^7^ A global survey conducted by the WHO in 2002 revealed a lack of attention to geriatrics in UG medical curricula.^5^ It was also disconcerting that data from only one sub-Saharan African (SSA) country, namely Ghana, had been included in that study. A 2015 survey of 25 medical schools across 11 countries in Africa reported on inclusion of geriatric topics at UG level in only 60% of the participating institutions.^8^ Dedicated teaching time for geriatrics was very limited, consisting of < 10 h in relation to the entire degree programme. Furthermore, < 30% of the medical schools surveyed in SSA included examinable learning objectives in geriatrics. The main factor identified for the limited inclusion of geriatrics in UG medical curricula in SSA medical schools was the absence of a national curriculum.^5^ The low priority afforded to geriatric training at UG medical level was reportedly because of limited space in the curriculum, scarcity of teaching faculty in geriatrics and low levels of interest by staff and students.^9^

Most initiatives to prepare medical graduates to care for geriatric patients are from high-income countries (HICs), which have markedly different resources and health systems than those of SSA. Professional bodies in HIC such as the American Geriatric Society, British Geriatric Society and Australian Society for Geriatric Medicine have advocated for the inclusion of geriatric medicine in the UG medical curriculum of their countries.^10,11,12^ These bodies of specialist geriatricians have developed and proposed minimum core competencies for geriatric medical education, many of which are derived from recommendations of the International Association of Gerontology and Geriatrics (IAGG), a non-governmental organisation that aims to promote training in geriatric care globally (Online Appendix 1).^10,11,12,13,14,15^ Although the IAGG recommendations are no longer available online, the principles of geriatric training for medical students remain largely the same. Medical educational reforms worldwide have focussed on a competency- or outcomes-based approach within the context of population needs.^16^ The development and adoption of minimum core competencies for geriatric care in national curricula have resulted in significant improvements in UG medical education in geriatrics in HICs.^17^

South Africa has, however, few specialist geriatricians and limited input from special interest groups towards prescribed geriatric training at UG medical level in South Africa.^9,18^ Furthermore, the Health Professions Council of South Africa (HPCSA), the body that regulates and accredits the training of health professionals in South Africa, does not have a prescribed national curriculum for UG medical training. Instead, the HPCSA has adopted a revised Canadian Medical Education Directions for Specialists (CanMEDS) framework to develop and assess seven attributes of medical graduates in the curricula.^19^ This core competency framework originally developed for physicians in the 1990s by the Royal College of Physicians and Surgeons of Canada has been refined and adopted by the HPCSA to guide a competency-based education approach for medical curricula. Each of the nine medical schools in the country has total autonomy over its UG medical programme, subject to accreditation by the HPCSA. A lack of attention to core minimum standards has resulted in either the omission or selective coverage of examinable geriatric competencies at UG level at South African medical schools.^20^ It is crucial that both local health systems and the curricula of medical schools represent and address the needs of the local population. This will ensure that medical schools are socially accountable and responsive to those communities being served by its graduates.^21,22^

Several studies conducted in South Africa report that geriatric patients in South Africa have many negative perceptions of health professionals and the care that they received.^23,24,25^ A recent study conducted in KwaZulu-Natal documented that geriatric patients described health care professionals as uncaring and lacking in respect for their elderly patients.^23^ Health services to older adults were perceived as ‘disease-centred’ and fragmented. These studies indicate the need to improve geriatric care training of health professionals, especially regarding behavioural and attitudinal attributes. There is strong evidence that patient-centred care, in particular, is highly valued by older patients and is essential for the management of complex health issues in older age.^23,24^ Curricula review is critical to determine how geriatric knowledge, skills and attitudes are addressed at UG medical level, and how medical educators can enhance current offerings to be more socially accountable.

Given the limited literature available on geriatric training for UG medical students in SSA, this study was undertaken to map the geriatric curriculum offered at the University of KwaZulu-Natal (UKZN) and identify inclusion of core geriatric competencies for medical graduates to attain. As proposed by Harden, this curriculum mapping study was conducted with a view to gaining insight into the content, teaching strategies and assessment methods relevant to the care of elderly patients.^26^ This will provide a benchmark of current teaching and assessment of geriatric-related learning objectives and provide part of the overall review of the geriatric curriculum at the UKZN.

## Methods

### Study design

This was a descriptive exploratory mixed methods study.

### Setting

This study was conducted at the UKZN, one of nine medical training facilities in South Africa where the undergraduate medical (MBChB) curriculum spans 6 years. Teaching in pre-clinical years of the programme follows a problem-based learning (PBL) approach, which exposes students to theoretical paper-based patient cases to stimulate their learning. There is a greater focus on clinical medicine in the latter 3 years of the programme. Geriatric topics and teaching were introduced into the UG medical curriculum in 2001, the same year that the UKZN appointed a chair in the Department of Geriatric Medicine.

### Data collection

A document review of the curriculum was undertaken, and semi-structured interviews were conducted with a purposive sample of health professions educators (*n* = 5). The participants were lecturers or tutors involved in geriatric-related teaching and curriculum development and were from the professions of family medicine, internal medicine, anatomical pathology and psychiatry.

Ethical approval was obtained prior to data collection from the UKZN Biomedical Research Ethics Committee. Data collection occurred between April and August 2019.

Learning objectives relevant to geriatric care were identified through a search on the web-based curriculum platform LOOOP (Learning Opportunities, Objectives and Outcomes Platform).^27^ This electronic platform contains information on all modules offered in the undergraduate medical curriculum and outlines individual learning objectives and related teaching and assessment. In addition, the contribution of the modules to each of the competency domains that medical doctors should master (CanMEDS competencies) is tabulated.^19^ Additional data were obtained from student and facilitator study guides, and semi-structured interviews with key informants. Interviews were audio-recorded and transcribed.

### Analysis

Learning objectives relevant to the care of older adults were extracted from LOOOP, collated on an Excel spreadsheet and categorised according to the year of study as documented in Online Appendix 1. A summary of the information regarding the teaching and assessment methods used for each domain in geriatric care is reported per academic year of study as indicated in Table 1. The qualitative data from the interviews with the health professions educators (*n* = 5) were analysed for content that contributed to the study objectives.

**TABLE 1:** Domains of geriatric care taught and assessed in the MBChB curriculum.

No.	Geriatric topic	Year	Learning	Assessment
1	Principles of geriatrics	1	Lecture	MCQ
2	Prescribing for the elderly	3	Lecture	MCQ, OSPE
3	Legal and ethical issues of ageing	3	Lecture	MCQ, OSPE
4	Physiological changes of ageing	3	Lecture	MCQ, OSPE
5	Dementia – risk factors, assessment and management	3,4,5,6	Lectures	MCQ, OSPE, case study(p), long case†, DOSCE
6	Comprehensive Geriatric Assessment (CGA)	3,4,5,6	Lectures, clinical	MCQ, OSPE, case study(p), long case†, DOSCE
7	Urinary incontinence	3,4,5,6	Lectures, clinical	MCQ, OSPE, case study(p), long case†, DOSCE
8	Falls	3,4,5,6	Lectures, clinical	MCQ, OSPE, case study(p), long case†, DOSCE
9	Infections in the elderly	3,4,5,6	Lectures, clinical	MCQ, OSPE, case study(p), long case†, DOSCE
10	Frailty	3,4,5,6	Lectures, clinical	MCQ, OSPE, case study(p), long case†, DOSCE
11	Confusion	3,4,5,6	Lectures, clinical	MCQ, OSPE, case study(p), long case†, DOSCE
12	Syncope	3,4,5,6	Lectures, clinical	MCQ, OSPE, case study(p), long case†, DOSCE
13	Osteoporosis	3,4,5,6	Lectures, clinical	MCQ, OSPE, case study(p), long case†, DOSCE
14	End-of-life care (palliative care)	5	Lectures, small group seminars	Case study (p), MCQs
15	Geriatric psychiatry – neurocognitive disorders	6	Lectures, clinical	MCQs, OSCE, case studies (p)

MCQ, multiple-choice questions; OSPE, objective structured practical examination; OSCE, objective structured clinical examination; DOSCE- directly observed clinical assessment, (p), portfolio of evidence for assessment.

† , Long case – Assessment of long clinical case.

### Ethical considerations

Ethical clearance was obtained from the University of KwaZulu-Natal Biomedical Research Ethics Committee (BE287/18).

## Results

### Geriatric content

There were 15 domains of geriatric care training identified in the curriculum, as tabulated in Table 1. Common conditions among geriatric patients such as urinary incontinence, falls, infections, dementia, frailty, confusion, syncope and osteoporosis were formally taught and assessed. Geriatric clinical skills such as the Comprehensive Geriatric Assessment (CGA)^28^ and Mini-mental State Examination (MMSE)^29^ were also taught to students. The CGA is an evaluation conducted by a multidisciplinary team to determine an elderly person’s medical, psychosocial, functional and environmental resources and problems. This is linked with a coordinated plan to improve overall patient functioning and independence.

### Teaching methods in geriatric topics

The geriatric content is delivered through a total of 40 h of didactic lectures and a few case discussions, and approximately 10 h of practical or bedside teaching mainly to students in years 4–6. Additional resources including journal articles serve as electronic resources for self-directed learning among students. Formal teaching of communication skills, as offered at the first- and second-year level, did not include strategies to address the challenges of communicating with elderly patients.

Clinical teaching in geriatrics occurred mainly at academic hospitals and was predominantly achieved during the internal medicine modules, with some coverage in psychiatry and family medicine. The limited involvement of other disciplines to include teaching in geriatrics was highlighted by a comment from a geriatric teacher:

‘… there is no interest from the other divisions to include geriatrics. For instance, in cardiology there is discussion on how it develops from childhood to adulthood, but there is little emphasis on changes from adulthood into old age.’ (Participant A, female senior lecturer, more than 20 years experience, Clinical Medicine Department)

The review of the LOOOP platform did not reflect any planned bedside teaching around geriatric topics as these occurred infrequently and depended on the availability of geriatric inpatients. Bedside teaching, when it did occur, was not standardised. The programme did not include clinical teaching on ambulatory or community-dwelling older adults despite the awareness of some of the teachers of the benefits of this exposure to students’ learning:

‘Students need to go out and see geriatric patients. That’s what really sticks with students.’ (Participant B, male senior lecturer > 20 years, Family Medicine)

Another participant expressed concern about the lack of an integrated patient-centred approach to geriatric patients as currently practised in the teaching in separate disciplines:

‘The assessment of the geriatric patient is so patient-centred. Each individual patient differs so much in the way you approach them.’ (Participant C, female clinical tutor > 10 years, Family Medicine)

Apart from a single lecture being offered by occupational therapy on the management of dementia, almost all the lectures are delivered by a specialist geriatrician or psychiatrist. The current programme includes neither the use of multidisciplinary teams nor interprofessional educational strategies in delivering the geriatric curriculum.

### Assessment methods

The assessment of geriatric content in years 1 to 3 forms part of the integrated module assessment and no sub-minima are applied to the geriatric content. The assessment of geriatric knowledge and skills contributes to approximately one-tenth of the internal medicine assessment of students in years four, five and six, also with no sub-minima. Assessment methods include multiple-choice questions, clinical examination, objective structured clinical examinations (OSCE), directly observed clinical assessment (DOSCE) and the assessment of portfolios of learning, as listed in Table 1. All the assessment methods are targeted at assessing students’ geriatric knowledge and skills, and none examine attitudinal components of geriatric competencies.

### Inclusion of recommended geriatric competencies

**FIGURE 1:**
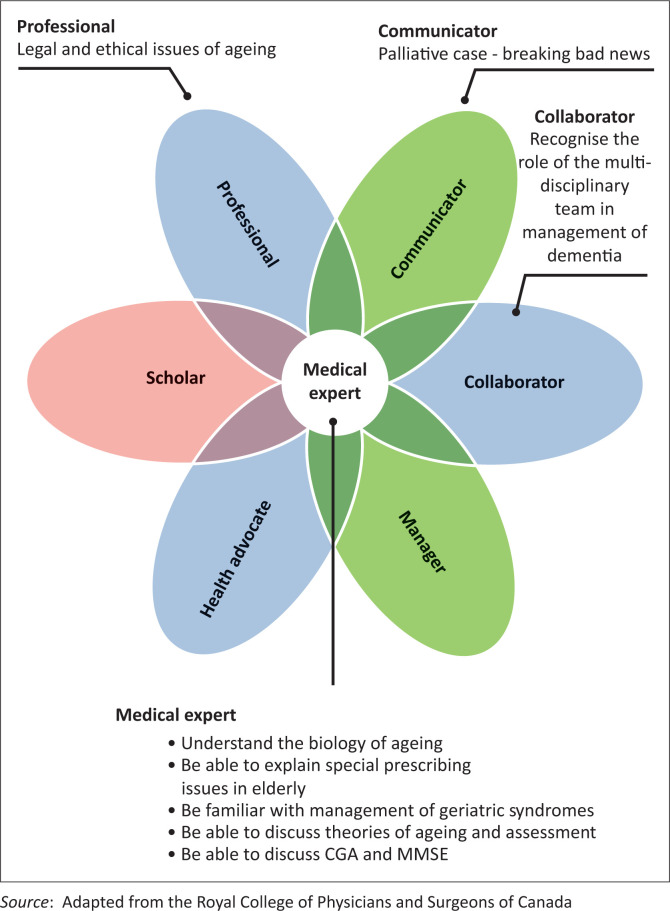
Mapping geriatric competencies as interpreted through the CanMEDS framework.

The domains of geriatric care taught and assessed were related to each of the seven graduate roles in the CanMEDS framework, as depicted in Figure 1. The central role of the ‘medical expert’ integrates the other six roles of ‘collaborator’, ‘communicator’, ‘health advocate’, ‘manager’, ‘professional’ and ‘scholar’. Learning objectives relevant to geriatric care contributed to four of the seven graduate attributes outlined in the CanMEDS framework (Figure 1). There were no learning objectives in the geriatric care that specifically contributed to graduate attributes of scholar, health advocate and manager (Figure 1).

## Discussion

This study indicates that the UG medical curriculum at UKZN includes a wide range of examinable learning objectives relevant to the care of older patients. The programme offers good coverage of key geriatric topics including geriatric syndromes and CGA. This is possibly because of the presence of a dedicated department of geriatric medicine to drive teaching and learning.^30^ Although the total contact time for teaching geriatric-relevant topics at UKZN was greater than that reported by Frost et al. in other SSA medical schools, the proportion of teaching time in geriatrics is still small in relation to the entire programme.^8^ The curriculum coverage does not adequately represent the high demand for health care services by older adults in relation to the general population.

Most of the teaching is furthermore concentrated in the last 3 years of the curriculum, with only a few lectures and seminars in the first and third years. Studies have shown that early exposure to geriatrics improves both knowledge as well as attitudes of medical students regarding care of elderly patients.^31,32,33^ Learning opportunities for students in the early years could include communication skills with older adults as well as some of the IAGG-recommended competencies not currently included in the curriculum.^34^

The methods of teaching are mostly classroom based with emphasis on students’ acquisition of knowledge. There is limited clinical exposure to geriatric patients in hospital settings, who represent only a small segment of the population requiring medical care. Most of the medical care to geriatric patients is delivered at primary care level, among whom the burden of disease is higher than in any other age group because of the high prevalence of chronic diseases and multi-morbidity. Exposure to geriatric inpatients with complex health problems has been shown to reinforce negative stereotypes about older patients and adversely affect students’ attitudes towards caring for elderly patients.^35^ It is essential that teaching in geriatric care be expanded to more settings to ensure greater teaching and learning concordance in authentic contexts where graduates are most likely to encounter elderly patients. Students require interaction with ambulant and community-dwelling older adults to appreciate the complexities of healthcare in the aged and develop a patient-centred approach. Exposure to well elderly people in the community as practised in the Senior Mentor programme in some medical schools in the USA promotes student learning in geriatrics, as well as positive attitudes towards caring for older adults.^36^

The current programme neither facilitates interaction between medical and other health profession students nor involves interprofessional education. Many of the IAGG core competencies in geriatric care require an understanding of the role of the multi-disciplinary team in the care of geriatric patients. Although there is some inclusion of teaching by occupational therapy on the management of dementia, there is little reinforcement of that teaching elsewhere in the curriculum. The ability to function in a multi-disciplinary team is particularly important in the management of geriatric patients where the aim is to preserve function and quality of life.^37^ Interprofessional education has also been shown to be effective in improving student attitudes towards patients and other health professionals, as well as align medical education with patient-centred care.

Regarding the assessment of geriatric learning objectives, our study found that only students’ geriatric knowledge and skills were assessed and not their attitudes towards caring for elderly patients. To assess students’ attitudes towards care of older patients, appropriate tools need to be developed and validated. Current assessment of geriatric topics contributes only a small component to overall assessments, with no sub-minima. There is thus insufficient evidence to determine the actual competencies of medical graduates in geriatric care. Separate assessment of geriatric components in UG medical curricula has been shown to improve both student knowledge and attitudes in geriatric care.^38^ However, the feasibility of this model for the SSA context will have to be explored, especially given the context of overcrowded health care curricula. Introducing a sub-minimum in the assessment of geriatric topics would at least simulate student learning in those domains of geriatric care that is taught.

Our analysis of the curriculum showed that only geriatric knowledge and skills are targeted, and student attitudes are not addressed. In addition, the medical graduate roles of scholar, manager and health advocate regarding geriatric care were not addressed. This omission may partly be responsible for the low priority afforded to the health needs of older patients in South Africa. In view of the negative reports regarding poor attitudes of health professionals towards elderly patients, it is essential that development and assessment of professional attitudes are an explicit part of the curriculum. The findings of this study highlight the need for consensus by relevant stakeholders on the minimum medical graduate competencies in geriatric care to guide medical curriculum planners. This will ensure that all medical graduates possess the minimum competencies necessary to care for their older patients.

Because of the low priority afforded to geriatrics in the curriculum, students may perceive the discipline as unimportant and fail to attain essential competencies necessary to care for the elderly patients. Similar to the findings reported on the geriatric curricula of other health professions trained at UKZN, this study identified a need for a policy to inform curriculum development for health professions training in geriatric care.^39^ The next step of the curriculum review framework^40^ suggests a targeted needs assessment of the local geriatric community and an evaluation of the outcome of the current geriatric curriculum on student knowledge, attitudes and perceptions regarding medical care of elderly patients. This would inform educational guidelines for UG medical and other health professions training in geriatric care.

### Study limitations

This study was conducted at a single institution, and therefore the findings may not be generalisable to other medical curricula. This study only examined the planned and delivered geriatric curriculum as captured on the LOOOP platform as of July 2019. Data from student manuals and participants highlighted that some teaching activities, such as the case discussions, were not recorded on the electronic platform. Hence, teaching and learning not documented may have been omitted from this analysis. This study also did not explore the ‘hidden’ curriculum in geriatrics. This is the unwritten and unintended lessons and perspectives that students learn in the educational environment.^41^

## Conclusion

This study evaluated the geriatric curriculum at the UKZN, and the findings provide a benchmark for comparison with the curricula at other medical schools in SSA. The presence of a department of Geriatric Medicine at the UKZN has helped to drive and implement teaching and assessment of key learning objectives in geriatric care. However, geriatric teaching faculty are in short supply, and innovative strategies are required to enhance geriatric teaching that is relevant to the SSA context. These should include interprofessional education and community partnerships, the importance of which has been highlighted in this study.

Consensus over a national core geriatric competency list is needed to standardise health professions training in geriatric care. It will require greater stakeholder involvement from professional bodies and geriatric communities to ensure that there is adequate representation of geriatric competencies in the UG curriculum. Consensus on the core curriculum will also inform discussion about suitable early and longitudinal student exposure and the educational settings that will result in the development of appropriate attitudes in medical graduates.

This study forms part of an internal curriculum review process. Further evidence is needed on the outcome of the curriculum on medical student knowledge and attitudes regarding the care of geriatric patients, and the feasibility of interprofessional education models for geriatric care training of health professions students. This will inform the development of educational guidelines for UG medical education in geriatric care at the UKZN.
